# Calponin 3 Regulates Myoblast Proliferation and Differentiation Through Actin Cytoskeleton Remodeling and YAP1-Mediated Signaling in Myoblasts

**DOI:** 10.3390/cells14020142

**Published:** 2025-01-18

**Authors:** Mai Thi Nguyen, Quoc Kiet Ly, Thanh Huu Phan Ngo, Wan Lee

**Affiliations:** 1 Department of Biochemistry, Dongguk University College of Medicine, 123 Dongdae-ro, Gyeongju 38066, Republic of Korea; nguyenmainhp@gmail.com (M.T.N.); kietly1501@gmail.com (Q.K.L.); thngo139@gmail.com (T.H.P.N.); 2 Section of Molecular and Cellular Medicine, Medical Institute of Dongguk University, Dongguk University College of Medicine, 123 Dongdae-ro, Gyeongju 38066, Republic of Korea; 3 Channelopathy Research Center (CRC), Dongguk University College of Medicine, 32 Dongguk-ro, Ilsan Dong-gu, Goyang 10326, Republic of Korea

**Keywords:** calponin, actin cytoskeleton remodeling, myogenic differentiation, YAP1, mechanotransduction, proliferation

## Abstract

An actin-binding protein, known as Calponin 3 (CNN3), modulates the remodeling of the actin cytoskeleton, a fundamental process for the maintenance of skeletal muscle homeostasis. Although the roles of CNN3 in actin remodeling have been established, its biological significance in myoblast differentiation remains largely unknown. This study investigated the functional significance of CNN3 in myogenic differentiation, along with its effects on actin remodeling and mechanosensitive signaling in C2C12 myoblasts. CNN3 knockdown led to a marked increase in filamentous actin, which promoted the nuclear localization of Yes-associated protein 1 (YAP1), a mechanosensitive transcriptional coactivator required for response to the mechanical cues that drive cell proliferation. Subsequently, CNN3 depletion enhanced myoblast proliferation by upregulating the expression of the YAP1 target genes related to cell cycle progression, such as cyclin B1, cyclin D1, and PCNA. According to a flow cytometry analysis, CNN3-deficient cells displayed higher S and G2/M phase fractions, which concurred with elevated proliferation rates. Furthermore, CNN3 knockdown impaired myogenic differentiation, as evidenced by reduced levels of MyoD, MyoG, and MyHC, key markers of myogenic commitment and maturation, and immunocytochemistry showed that myotube formation was diminished in CNN3-suppressed cells, which was supported by lower differentiation and fusion indices. These findings reveal that CNN3 is essential for myogenic differentiation, playing a key role in regulating actin remodeling and cellular localization of YAP1 to orchestrate the proliferation and differentiation in myogenic progenitor cells. This study highlights CNN3 as a critical regulator of skeletal myogenesis and suggests its therapeutic potential as a target for muscle atrophy and related disorders.

## 1. Introduction

Skeletal muscle comprises approximately 50% of total body mass and is essential for various physiological processes, including locomotion, respiration, and metabolism [1]. The process of muscle tissue formation and regeneration, referred to as myogenesis, plays a vital role in muscle mass maintenance, especially in response to disease or injury [2,3]. Myogenesis involves a sequence of cellular events that begin with myoblast proliferation, followed by myoblast differentiation into myocytes, and culminating in the fusion of myocytes into multinucleated myotubes, which ultimately form functional muscle fibers [4,5]. Skeletal myogenesis is meticulously coordinated by an intricate signaling network, with cytoskeletal dynamics that are particularly critical for myoblast differentiation, fusion, and maturation [6,7]. Recent research has highlighted that mechanotransduction is a pivotal regulatory mechanism in skeletal myogenesis, which involves the transduction of mechanical cues into biochemical signals for the myogenic transcription program [8,9]. Accordingly, dysregulation in cytoskeletal remodeling has been directly associated with defects in myoblast differentiation, thereby impeding the formation of myotubes from progenitor cells [10,11,12,13].

The actin cytoskeleton is especially dynamic and undergoes precise spatial and temporal remodeling, which supports the structural and functional alterations required for myogenesis [14,15]. This remodeling is facilitated by various actin-binding proteins (ABPs) that coordinate actin assembly, disassembly, and stability to support rapid cellular adaptations [15,16]. Among these ABPs, calponin 3 (CNN3), a member of the calponin family, modulates cytoskeletal rearrangement, stress fiber formation, and actomyosin interactions through its actin-binding ability [17,18]. Therefore, CNN3 plays a pivotal role in actin cytoskeleton remodeling and maintaining cytoskeletal integrity [18,19,20]. Previous studies have shown that CNN3 is expressed in myoblasts and is critical for actin stabilization during trophoblast fusion in embryonic development [18,21]. Muscle atrophy, including sarcopenia and cachexia, is marked by impaired myogenic differentiation and diminished muscle regeneration [3,22]. Recent evidence further indicates that CNN3 levels are diminished in various muscle atrophy models, such as starvation-induced myotube atrophy in murines [23] and tibial muscular dystrophy in humans [24]. Thus, it appears that CNN3 likely plays a crucial role in muscle development and preservation, and its reduction might impair myogenesis and contribute to muscle atrophy. Despite its established role in actin dynamics, the specific functions of CNN3 in skeletal myogenesis, particularly regarding myogenic differentiation and myotube formation, remain largely unexplored.

Emerging evidence suggests that mechanotransduction pathways, particularly those mediated by actin dynamics, are central to determining cellular fate by modulating transcriptional programs and cell cycle progression [25]. In this aspect, the balance between monomeric globular actin (G-actin) and polymerized filamentous actin (F-actin) is integral to these pathways by controlling the cellular localization of Yes-associated protein 1 (YAP1), which is a mechanosensitive transcriptional coactivator that is essential for mediating cellular responses to mechanical stimuli [26,27]. In response to elevated F-actin levels, YAP1 translocates to the nucleus, facilitating cell cycle transition and proliferation [28]. This mechanism ultimately suppresses the differentiation of progenitor cells by maintaining them in a proliferative state rather than allowing them to transition into mature myogenic cells [4,5]. Thus, this F-actin-driven YAP1 activation links cytoskeletal dynamics to the suppression of myogenesis by driving the expressions of the genes associated with proliferation [26,27,29]. Given the role played by CNN3 in actin remodeling, it is likely involved in modulating mechanosensitive YAP1 signaling during myogenic differentiation. However, the precise function of CNN3 has yet to be fully clarified, and understanding its contributions could offer significant insights into muscle development and regeneration mechanisms.

This study aimed to elucidate the role of CNN3 in myogenic differentiation by demonstrating its influence on actin remodeling and key mechanosensitive signaling pathways. Specifically, we examined the effects of CNN3 knockdown on actin remodeling, YAP1 nuclear localization, modulation of cell cycle phases, and cell proliferation, as well as its downstream impact on the expression of myogenic factors and the transition from myoblasts to myotubes. This research identifies CNN3 as a vital regulator of myoblast proliferation and differentiation, operating through its modulation of actin dynamics and the mechanosensitive transcriptional program.

## 2. Materials and Methods

### 2.1. Mouse Tissue Sample

Various tissues were obtained from an acclimated 8-week-old male C57BL/6 mouse following euthanasia by cervical dislocation. The in vivo experimental protocol was approved by the Animal Care and Use Committee of Dongguk University (Approval No. IACUC-2021-009). Tissues were washed with ice-cold saline and processed using a tissue homogenizer (Omni International, Inc., Kennesaw, GA, USA). Immunoblot analysis was performed on 10 µg of protein from each tissue sample using anti-CNN3 and β-actin antibodies.

### 2.2. Cell Culture

C2C12 myoblasts (CRL-1772, ATCC, Manassas, VA, USA) were cultured in a growth medium (GM) composed of DMEM containing 10% fetal bovine serum (FBS) and 100 units/mL streptomycin/penicillin (Gibco, Carlsbad, CA, USA). For myogenic differentiation, the cells were seeded in 35 mm dishes (~1.3 × 10⁵ cells per dish) and maintained in GM until they reached approximately 90% confluency. Differentiation was then initiated by transitioning to a differentiation medium (DM; DMEM supplemented with 2% horse serum (Gibco)). Cells were incubated in DM for up to five days with medium changes every 24 h.

### 2.3. Oligonucleotide Transfection

C2C12 cells were seeded in 35 mm dishes at a density of approximately 1.3 × 10⁵ cells per dish and allowed to reach 40–50% confluency over 20–24 h. Transfection was performed using 200 nM of scrambled control RNA (scRNA, Genolution, Seoul, Republic of Korea), CNN3 siRNA (siCNN3-1, Bioneer, Daejeon, Republic of Korea), or an alternative CNN3 siRNA (siCNN3-2, Genolution) with Lipofectamine 2000 (Invitrogen, Waltham, MA, USA) in serum-free DMEM for 4 h. Cells were subsequently cultured in GM for 24 h. The sequences of the oligonucleotides are provided in Table S1.

### 2.4. RT-qPCR

RNA was extracted from the cells using a Total RNA Miniprep kit (Enzynomics, Daejeon, Republic of Korea) and quantified using a nanodrop spectrophotometer (Keen Innovative Solutions, Daejeon, Republic of Korea). cDNA was generated using the miScript II RT Kit (Qiagen, Hilden, Germany). Relative mRNA levels were assessed via RT-*q*PCR using SYBR Green (Enzynomics) in a LightCycler 480 (Roche Applied Science, Basel, Switzerland). Reactions were performed in triplicate, and the expression levels were normalized versus GAPDH using the 2^–ΔΔCT^ method. Details of the primer sequences and reaction conditions are provided in Table S2.

### 2.5. Cytoplasmic and Nuclear Fraction Extraction

Cells were collected with EDTA/trypsin (Gibco) 24 h after transfection, and cytoplasmic and nuclear fractions were separated using NE-PER Reagents (Thermo Fisher Scientific), in accordance with the manufacturer’s instructions. Cells were incubated on ice with CER I solution for 10 min, and then, CER II solution was added. Incubation continued for one min. Lysates were centrifuged at ~15,000 rpm for 15 min at 4 °C. The supernatants, corresponding to the cytoplasmic fractions, were collected, while the remaining pellets were resuspended in NER solution and centrifuged to isolate the nuclear fractions. Equal amounts of each fraction were then subjected to immunoblotting.

### 2.6. Immunoblotting

Cellular proteins were extracted using a lysis buffer composed of PBS supplemented with 2% Triton-X, 0.2 mM PMSF, and 1% phosphatase inhibitor cocktail II (Sigma-Aldrich, St. Louis, MO, USA). Protein concentrations were determined using the Bradford assay, and the samples were denatured by heating at 100 °C for 10 min. Proteins were separated by SDS-PAGE and subsequently transferred onto nitrocellulose membranes (Amersham Biosciences, Piscataway, NJ, USA). The membranes were blocked with 5% skimmed milk in TBST (TBS containing 0.5% Tween 20) for 1 h and incubated overnight at 4 °C with primary antibodies. The next day, the membranes were washed and incubated with the secondary antibodies diluted at 1:10,000. Blots were visualized using TOPviewTM ECL Femto Western Substrate (Enzynomics) and analyzed with Fusion Solo software (Paris, France). Details of the antibodies and their dilutions are provided in Table S3.

### 2.7. Immunocytochemistry

Cells were fixed with 4% paraformaldehyde for 10 min, permeabilized using 0.3% Triton X-100 for 15 min, and blocked with 3% BSA in PBS at room temperature for 2 h. They were subsequently incubated overnight at 4 °C with an anti-myosin heavy chain (MyHC) antibody diluted to 1:100. After washing, the samples were incubated with Alexa 488-conjugated secondary antibody (Invitrogen) for 1 h and counterstained with Hoechst 33,342. Using a Leica fluorescence microscope (Microsystems, Mannheim, Germany), images were obtained from five randomly selected fields. Using the ImageJ program (version 1.5.4), the nucleus numbers in myotubes, myotube diameters, and MyHC-positive areas were determined and subsequently calculated as previously described [12]. Briefly, the differentiation index was calculated as the ratio of MyHC-positive nuclei within the myotubes to the total number of nuclei in a given field. The fusion index was determined by measuring the percentage of myotubes with three or more nuclei relative to the total nuclei count. To ensure reliable statistical analysis, a minimum of five randomly chosen areas from three independent cultures were evaluated for each parameter. Experiments were conducted with a minimum of three independent replicates. F-actin was stained with FITC-conjugated phalloidin (Sigma) and analyzed using the ImageJ program.

### 2.8. Cell Proliferation Assay

Cell proliferation was measured using the Click-iT™ EdU Kit (Invitrogen). Briefly, C2C12 cells were seeded in 8-chamber slides at 3 × 10⁴ cells/well and transiently transfected with scRNA or siCNN3. At 24 h post-transfection, the cells were treated with 10 µM EdU for 4 h at 37 °C, then fixed with 4% paraformaldehyde, permeabilized with 0.3% Triton X-100 in PBS, and incubated with a Click-iT reaction cocktail. The nucleus was counterstained with Hoechst 33,342, and fluorescent images were captured using a Leica fluorescence microscope. Total and EdU-positive cell numbers were quantified in five randomly selected fields using the ImageJ program, and experiments were performed independently at least three times.

### 2.9. Cell Viability

Myoblasts were seeded in 96-well at a density of 10³ cells per well and transfected with siRNA or siCNN3 using Lipofectamine 2000 (Invitrogen). Cells were then incubated in GM containing 10 µL of Cell Viability Assay solution (Quanti-Max WST-8, BioMax, Seoul, Republic of Korea) for 4 h at 37 °C. Cell viability was evaluated by measuring absorbance at 450 nm using a microplate reader (Model 680, Bio-Rad, Hercules, CA, USA).

### 2.10. Flow Cytometry

Myoblasts were collected using trypsin/EDTA, centrifuged at 5000 rpm for 5 min at 4 °C, rinsed with PBS, fixed in 70% ethyl alcohol overnight at 4 °C, and stained for 20 min using the Propidium Iodide Flow Cytometry Kit (ab139418, Abcam, Cambridge, UK) for cell cycle analysis or FITC-phalloidin for F-actin quantitation. Samples were analyzed using a CytoFLEX instrument (Beckman Coulter, Brea, CA, USA).

### 2.11. Statistical Analysis

Results are expressed as means ± standard errors (SEM) from at least three independent experiments. One-way analysis of variance (ANOVA) was used to compare group differences. Post hoc analysis was conducted using Tukey’s test when significant differences were detected. A *p*-value below 0.05 was considered to be statistically significant.

## 3. Results

### 3.1. CNN3 Expression Is Upregulated During the Differentiation of Myoblast

**Figure 1:**
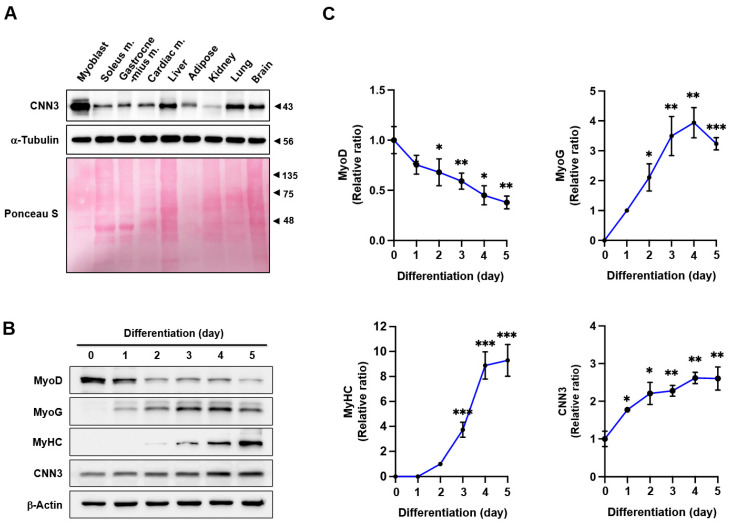
Expression of CNN3 in mouse tissues and throughout myoblast differentiation. (**A**) Immunoblotting was conducted to assess CNN3 expression in C2C12 myoblasts and tissues from 8-week-old mice; α-tubulin was used as a loading control. (**B**) C2C12 myoblasts were collected and subjected to immunoblotting of MyoD, MyoG, MyHC, and CNN3 at designated differentiation time points; β-actin was used as a loading control. (**C**) Expression levels were normalized versus β-actin, and relative ratios were calculated, with day 0 set as the baseline for MyoD and CNN3, day 1 for MyoG, and day 2 for MyHC. Data are expressed as means ± SEM (*n* = 3), with asterisks denoting statistically significant differences (* *p* < 0.05, ** *p* < 0.01, *** *p* < 0.001).

The differentiation of progenitor cells depends on the remodeling of the actin cytoskeleton, a process meticulously regulated by ABPs [15,16]. Recognizing the critical role of CNN3 in actin cytoskeleton dynamics [18,19,20,21], we first assessed its expression in mouse tissues, including myoblasts and skeletal muscles, prior to exploring its involvement in myoblast differentiation. As shown in Figure 1A, CNN3 was ubiquitously expressed significantly more in myoblasts than in the skeletal muscles that have fully developed, i.e., the soleus and gastrocnemius muscles. Additionally, the CNN3 expression was higher in liver, lung, and brain tissues compared to fully differentiated muscles. We also monitored its expression profile over the 5-day differentiation period in C2C12 myoblasts (Figure 1B,C). MyoD, a marker of myogenic commitment, showed a decline in expression following the onset of differentiation, whereas MyoG, a marker indicating the initiation of myogenic differentiation, exhibited a gradual increase, peaking on day 3 of differentiation. Myosin heavy chain (MyHC), a marker of terminal differentiation, displayed a steady rise starting on day 2, reaching its maximum expression after day 4 (Figure 1B,C). Interestingly, CNN3 expression increased as the differentiation process advanced toward myotube formation (Figure 1B,C), suggesting that CNN3 might play a role in regulating myogenic differentiation, possibly through the modulation of actin cytoskeletal dynamics and thereby influencing the myogenic transcriptional process via the mechanosensitive signaling pathway.

### 3.2. CNN3 Knockdown Increased F-Actin Levels and Nuclear Localization of YAP1 in Myoblasts

**Figure 2:**
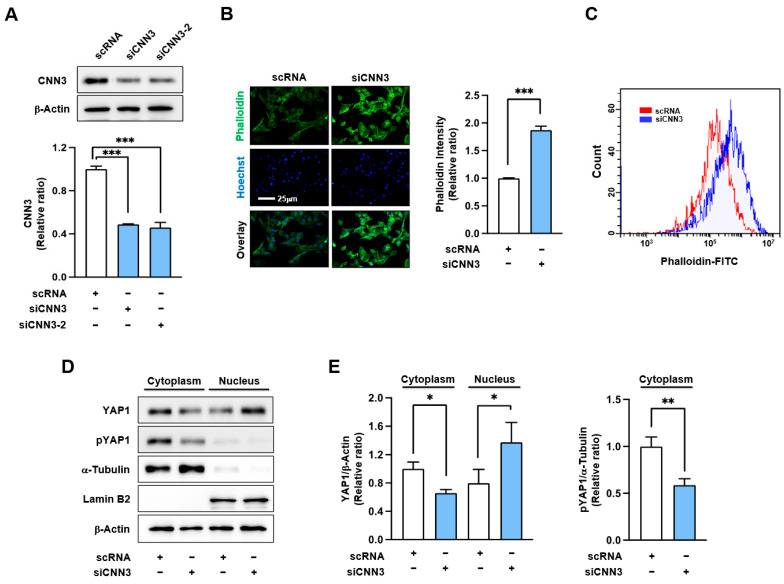
CNN3 knockdown enhanced F-actin levels and increased nuclear YAP1. C2C12 myoblasts were transfected with 200 nM of either control scRNA or siCNN3 (siCNN3-1 or siCNN3-2). (**A**) CNN3 expressions were determined 24 h after transfection by immunoblotting. Expression levels were normalized versus β-actin, and relative ratios were calculated using the control scRNA as the baseline (set to one). (**B**) Cells were stained with FITC-phalloidin (green) to visualize F-actin and Hoechst 33,342 (blue) to label nucleus. Scale bar: 25 μm. Phalloidin fluorescence intensities were quantified using ImageJ program. (**C**) F-actin levels were analyzed by flow cytometry following staining with FITC-phalloidin. (**D**) Cytoplasmic and nuclear fractions were subjected to immunoblotting to detect YAP1, pYAP1 (phosphorylated YAP1), and CNN3. α-Tubulin and lamin B2 were used as markers for the cytoplasmic and nuclear fractions, respectively; β-actin was used as a loading control. (**E**) Expression levels were normalized versus β-actin, and relative ratios were calculated versus scRNA. Data are expressed as means ± SEM (*n* = 3), with asterisks denoting statistically significant differences (* *p* < 0.05, ** *p* < 0.01, *** *p* < 0.001).

To determine whether CNN3 knockdown affects myogenic differentiation, we hypothesized that CNN3 suppression could alter actin filament remodeling and impair myogenic differentiation by promoting the nuclear localization of YAP1. To test this hypothesis, C2C12 myoblasts were transfected with control scrambled RNA (scRNA) or CNN3-specific siRNAs (siCNN3-1 or siCNN3-2) and cultured for 24 h in a GM. Both siRNA constructs resulted in an approximately 50% reduction of CNN3 protein compared to the controls (Figure 2A). Therefore, siCNN3-1 (briefly named siCNN3) was used to suppress the CNN3 protein level for subsequent experiments in this study.

Next, we evaluated whether CNN3 knockdown altered F-actin levels in myoblasts. Fluorescent phalloidin staining conducted 24 h post-transfection in myoblasts demonstrated that CNN3 knockdown significantly elevated F-actin levels by approximately 1.7-fold compared to the controls (Figure 2B). Flow cytometry further confirmed this observation by demonstrating elevated phalloidin fluorescence, which is indicative of increased actin polymerization (Figure 2C). As the actin levels remained unchanged 24 h after transfection with siCTTN (Figure 2D), the observed increase in F-actin was ascribed to reduced actin depolymerization caused by CNN3 suppression rather than an upregulation of actin expression. The accumulation of F-actin facilitates the localization of YAP1 in the nucleus by inhibiting its phosphorylation [30]. To investigate whether CNN3 knockdown affects the localization of YAP1, we measured YAP1 levels in the cytoplasmic and nuclear fractions of C2C12 myoblasts. Our results revealed a significant reduction in cytoplasmic YAP1 phosphorylation and cytoplasmic YAP1 levels, accompanied by an increase in nuclear YAP1 localization in siCNN3-transfected cells (Figure 2D,E). Notably, the total cellular YAP1 levels remained unchanged, indicating that siCNN3 inhibits YAP1 phosphorylation without altering its total expression (Supplementary Figure S1). These findings suggest that CNN3 knockdown enhances F-actin accumulation, leading to reduced YAP1 phosphorylation and promoting its nuclear translocation.

### 3.3. CNN3 Knockdown Increased YAP1 Target Gene Expressions and Induced Myoblast Proliferation

**Figure 3:**
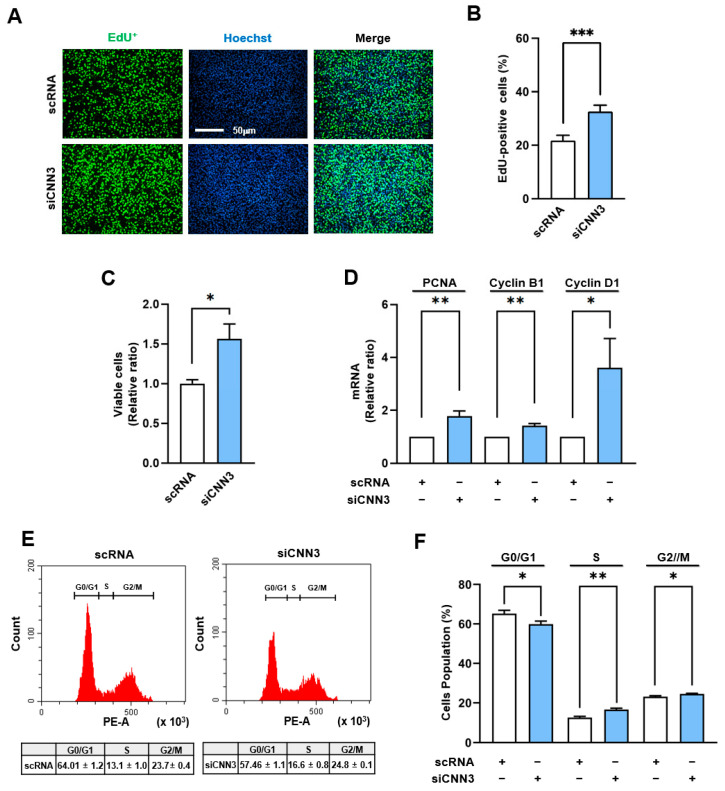
CNN3 depletion facilitated myoblast proliferation. C2C12 myoblasts were transfected with either control scRNA or siCNN3 and analyzed 24 h post-transfection. (**A**) Cell proliferation was assessed using EdU incorporation (green) to label replicating cells, and Hoechst 33,342 (blue) was used to counterstain the nucleus. Scale bar: 50 µm. (**B**) The percentages of EdU-positive cells were determined using ImageJ program. (**C**) Viable cell numbers were measured using a cell viability assay kit. (**D**) mRNA levels of proliferation markers (PCNA, Cyclin B1, and Cyclin D1) were assessed by RT-*q*PCR and normalized versus GAPDH expression. (**E**,**F**) Cell cycle analysis was performed using flow cytometry with scatter plots. Data are expressed as means ± SEM (*n* = 3), with asterisks denoting statistically significant differences (* *p* < 0.05, ** *p* < 0.01, *** *p* < 0.001).

As the increased localization of YAP1 in the nucleus drives cell cycle progression and stimulates cell proliferation [31], we investigated whether CNN3 knockdown could stimulate these processes in C2C12 myoblasts. To address this, we assessed the proliferation of C2C12 myoblasts following transfection with either scRNA or siCNN3. From the results of the EdU incorporation analysis and the viable cell count, CNN3 knockdown led to a marked increase in EdU-positive cell numbers and overall cell viability, indicating enhanced proliferation compared to the controls (Figure 3A–C). Furthermore, an RT-*q*PCR analysis revealed significant upregulations of the mRNA levels of YAP1 target genes, including those of the proliferating cell nuclear antigen (PCNA), cyclin B1, and cyclin D1, in siCNN3-transfected cells (Figure 3D). A flow cytometry analysis also supported these findings by showing increased proportions of cells in the S and G2/M phases, along with a reduction in the G0/G1 phase (Figure 3E,F). Together, these observations suggest that CNN3 knockdown stimulates myoblast proliferation by enhancing YAP1 signaling and promoting cell cycle progression.

### 3.4. CNN3 Was Required for Myogenic Differentiation

**Figure 4:**
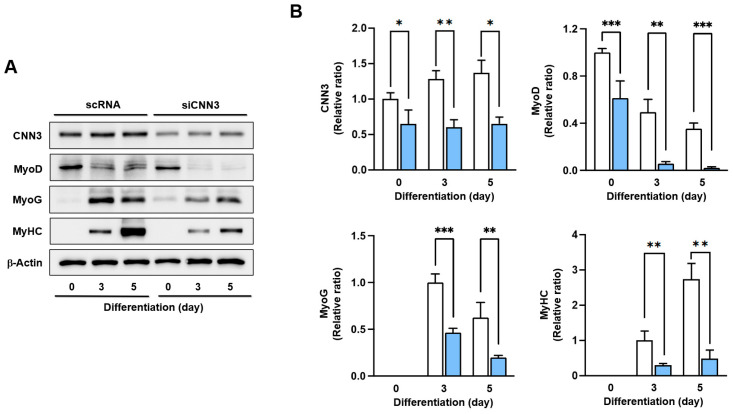
CNN3 knockdown suppressed expressions of myogenic factors. (**A**) C2C12 myoblasts were transfected with either control scRNA or siCNN3, allowed to differentiate, and subjected to immunoblotting at designated differentiation time points; β-actin was used as a loading control. Expression of myogenic regulatory factors and CNN3 were evaluated by immunoblotting. (**B**) Expression intensities of proteins in myoblasts transfected with scRNA (open column) and siCNN3 (blue column) were normalized versus β-actin. Results are expressed as relative ratios compared to scRNA levels on day 0 for CNN3 and MyoD and day 3 for MyoG and MyHC. Data are expressed as means ± SEM (*n* = 3), with asterisks denoting statistically significant differences (* *p* < 0.05, ** *p* < 0.01, *** *p* < 0.001).

Since increased cell cycle progression generally opposes progenitor cell differentiation [4], CNN3 depletion might suppress the expression of myogenic factors, thereby hindering myoblast differentiation. To test this hypothesis, we analyzed the expression of myogenic factors at differentiation days 0, 3, and 5 of C2C12 cells transfected with either scRNA or siCNN3. Our results revealed that the protein expression levels of MyoD, MyoG, and MyHC were significantly reduced in siCNN3-transfected cells compared to scRNA controls (Figure 4A,B). MyoD initiates myogenic differentiation by inducing MyoG expression, which subsequently drives myoblast differentiation [32]. The reduced levels of MyoG and MyHC further confirmed differentiation impairment. These results suggest that CNN3 is critical for initiating the expression of key myogenic regulatory factors, i.e., MyoD and MyoG, and is essential for proper myoblast differentiation.

**Figure 5:**
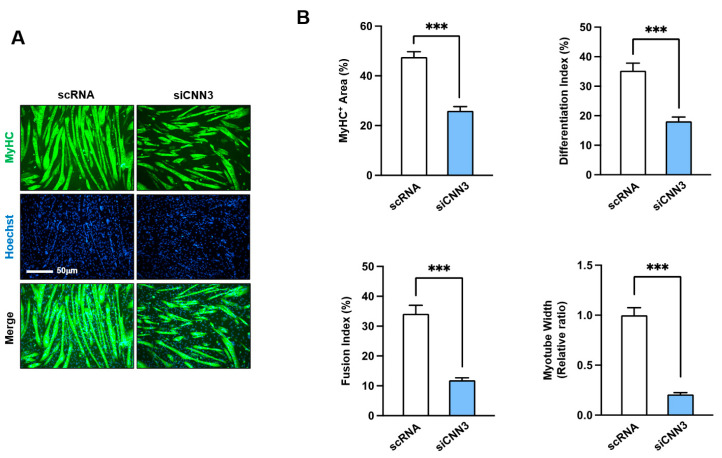
Depletion of CNN3 disrupted myogenic differentiation. C2C12 myoblasts were transfected with control scRNA or siCNN3 and then allowed to differentiate for 5 days. (**A**) Representative immunocytochemistry results after staining with MyHC antibody (green) and Hoechst 33,342 (blue). Scale bar: 50 μm. (**B**) MyHC-positive areas, differentiation and fusion indices, and myotube widths were evaluated as outlined in Section 2.7. Data are expressed as means ± SEM (*n* = 3), with asterisks denoting statistically significant differences (*** *p* < 0.001).

To further validate the functional significance of CNN3 in myogenic differentiation and myotube formation, immunocytochemical analyses of C2C12 cells were performed using a MyHC antibody at differentiation day 5. Myoblasts transfected with siCNN3 exhibited significantly impaired differentiation, as indicated by decreased MyHC-positive areas, lower differentiation and fusion indices, and reduced myotube width (Figure 5A,B). Collectively, these results underscore that CNN3 is crucial for proper myogenic differentiation and myotube formation; its absence inhibits myogenic regulatory factor expression, impairing myoblast fusion and maturation.

## 4. Discussion

Actin dynamics are fundamental to myogenesis, integrating mechanosensitive transcriptional programs with myogenic gene expression and cell cycle regulation to ensure the precise control of myogenic processes [9,15]. Although CNN3 has been shown to play a crucial role in actin cytoskeleton dynamics in various contexts [17,18], its function in the differentiation of progenitor cells during myogenesis has remained largely unknown. Previously, She et al. demonstrated that CNN3 is upregulated during myogenic differentiation and muscle regeneration, suggesting its role in regulating key pathways like AKT/mTOR and AMPK/mTOR, which are critical for muscle homeostasis and energy metabolism [33]. Building on this, our study elucidates an additional layer of CNN3′s regulatory roles in myogenesis by connecting its influence on actin remodeling and mechanosensitive transcriptional activation. Specifically, our findings demonstrate that (i) CNN3 expression is upregulated during differentiation, (ii) CNN3 knockdown elevates F-actin levels and promotes YAP1 nuclear localization, (iii) CNN3 depletion facilitates cell cycle progression and enhances myoblast proliferation, and (iv) CNN3 knockdown results in a marked decrease in the expression of myogenic regulatory factors and, thus, impairs differentiation, fusion, and myotube formation. These findings link CNN3 to the regulation of myogenic transcription factors and differentiation in myoblasts through the F-actin/YAP1 axis, a pathway previously unexplored in the context of CNN3. This study also unveils the pivotal role of CNN3 in coordination with myoblast proliferation and differentiation. The increased myoblast proliferation observed following CNN3 knockdown, accompanied by the suppressed expression of myogenic regulatory genes and impaired differentiation, indicates the essential role of CNN3 in coordinating cell cycle exit and initiating differentiation programs.

During myofiber formation, actin cytoskeletal remodeling is crucial for cellular morphology, membrane reconfiguration, and the activation of myogenic transcriptional programs [6,7]. We found that CNN3 expressions increase at the onset of differentiation and remain steady throughout myotube formation (Figure 1), implying its involvement in muscle development. Consistent with our findings, a recent study reported that CNN3 is upregulated during myogenic differentiation and muscle regeneration, suggesting its importance in these processes [33]. Furthermore, accumulating evidence has demonstrated the significance of CNN3 in embryonic myogenesis, where it is highly expressed in muscle tissues [18,21,34]. CNN3 regulates actin cytoskeleton rearrangement during embryonic development and promotes cell fusion in trophoblasts and myoblasts [18]. Moreover, CNN3 knockout caused embryonic and postnatal lethality, suggesting that it plays an essential role in development [21,34]. Together, these findings emphasize the essential role of CNN3 in facilitating key processes of myogenesis, mirroring its developmental functions reported in prior research.

We further demonstrated that CNN3 knockdown increases F-actin accumulation, potentially contributing to YAP1 nuclear localization in response to mechanical stress (Figure 2). Enhanced F-actin observed in CNN3-depleted myoblasts corresponds to the prior reports on fibroblasts and epithelial cells, where CNN3 depletion has been shown to promote actin polymerization [19,35]. Moreover, CNN3 depletion in lens epithelial cells similarly reorganized actin stress fibers, stimulated focal adhesion formation, and activated YAP1 signaling [36]. Our results involving CNN3 in myoblasts align with the findings of Nardone et al., who identified YAP as a key regulator of cell mechanics and focal adhesion assembly, suggesting its role as a mechanosensitive mediator in responding to cytoskeletal tension [37]. In progenitor cells, modifications to the cytoskeleton, such as actin polymerization/depolymerization and reorganization processes, are crucial for the regulation of the cell cycle and myogenic transcription program [14,38]. Hence, increased F-actin levels, as observed in CNN3-depleted cells, are known to inhibit Hippo signaling, a pathway that regulates YAP1 activity through phosphorylation by MST1/2 and LATS1/2 kinases [39,40]. Phosphorylation of YAP1 at key residues, such as Ser127, augments its retention in the cytoplasm by facilitating its interaction with 14-3-3 proteins, whereas reduced phosphorylation of YAP1 promotes its translocation into the nucleus [41]. Consequently, YAP1 acts as a transcriptional coactivator in the nucleus, binding to TEAD transcription factors and activating the genes associated with cell proliferation, survival, and tissue growth [26,27]. Thus, increased F-actin levels promote the nuclear localization of YAP1, driving cell proliferation and inhibiting differentiation by keeping progenitor cells in a proliferative state rather than facilitating their maturation into muscle cells [4,31]. CNN3 knockdown in myoblasts triggers these mechanisms by disrupting actin dynamics, leading to reduced YAP1 phosphorylation and subsequent nuclear translocation. Our findings indicate that CNN3 regulates YAP1 activity through its effects on F-actin dynamics rather than by direct interaction or a dystroglycan-like mechanism. This interpretation aligns with the role of CNN3 in maintaining cytoskeletal integrity and emphasizes the importance of actin remodeling in YAP1 localization. Although this study identified the impact of CNN3 on YAP1 nuclear translocation, the potential modulation by extracellular mechanical signals was not explored. Future research should examine how external mechanical cues influence the regulatory functions of CNN3 in myogenesis, providing a more comprehensive understanding of its role in mechanosensitive signaling.

Myoblast differentiation into myotubes throughout myogenesis requires an inverse relationship with proliferation, necessitating cell cycle arrest and the cessation of proliferation [4]. The temporal expression pattern of CNN3 during differentiation observed in this study (Figure 1) implies that CNN3 plays a significant role in exiting the proliferative cycle, thereby enabling cells to differentiate and fuse into myotubes. Indeed, CNN3 depletion markedly enhances myoblast proliferation, as demonstrated by increased cell viability, elevated EdU incorporation, and upregulated expression of cell cycle-related genes, including *PCNA*, *cyclin B1*, and *cyclin D1* (Figure 3). Furthermore, a flow cytometry analysis showed an increase in the proportion of cells in the S and G2/M phases in CNN3-depleted cells, indicating enhanced cell cycle progression (Figure 3). As described above, this increase in cell proliferation following CNN3 knockdown is causally linked to the nuclear localization of YAP1, which responds to the enhanced F-actin levels [28,42]. Recent studies have also established a connection between CNN3 expression and cell proliferation. For instance, an overexpression of CNN3 suppressed the invasion and proliferation of non-small cell lung cancer cells [43], whereas CNN3 knockout (-/-) embryos displayed increased proliferation of neuronal precursor cells [44]. Hence, the disrupted balance between proliferation and differentiation observed in CNN3-depleted myoblasts underscores the critical role of CNN3 in coordinating these processes during myogenesis. Consistent with the relation between proliferation and differentiation in progenitor cells, we further demonstrated that CNN3 knockdown suppresses the expression of key myogenic regulatory factors, such as MyoD, MyoG, and MyHC, thereby impairing myoblast differentiation and myotube formation (Figure 4 and Figure 5). Taken together, these findings establish a mechanistic link between F-actin accumulation, YAP1 activation, and the suppression of myogenesis in CNN3-depleted myoblasts. Thus, by regulating actin dynamics and the mechanosensitive YAP1, CNN3 plays a critical role in maintaining the balance between myoblast proliferation and differentiation.

## 5. Conclusions

This study identifies CNN3 as a key regulator of myoblast differentiation and proliferation due to its influence on actin cytoskeletal dynamics and the mechanosensitive transcription program for myogenic differentiation. CNN3 depletion leads to actin polymerization, stimulates the nuclear localization of YAP1, increases the expression of cell cycle-promoting genes, and consequently, provokes the downregulation of key myogenic regulatory genes to inhibit myoblast differentiation and myotube formation. This study identifies CNN3 as a critical regulator of skeletal myogenesis, offering valuable insights into the molecular mechanisms that govern muscle development and regeneration. By highlighting its role, the findings suggest CNN3 as a potential therapeutic target for muscle atrophy and related disorders. Further research into the regulation and function of CNN3 could deepen our understanding of muscle biology, providing a foundation for developing innovative therapies to prevent or treat muscle atrophy and associated conditions.

## Data Availability

The data presented in this study are available upon request from the corresponding author.

## Supplementary Materials

The following supporting information can be downloaded at https://www.mdpi.com/article/10.3390/cells14020142/s1, Table S1: Oligonucleotide sequences for transfection; Table S2: Primer lists and conditions for RT-*q*PCR; Table S3: Antibodies list; Figure S1: CNN3 knockdown did not alter YAP1 expression levels.

## References

[1] Brooks S.V., Guzman S.D., Ruiz L.P. (2023). Skeletal muscle structure, physiology, and function. Handb. Clin. Neurol..

[2] Schiaffino S., Dyar K.A., Ciciliot S., Blaauw B., Sandri M. (2013). Mechanisms regulating skeletal muscle growth and atrophy. FEBS J..

[3] Sartori R., Romanello V., Sandri M. (2021). Mechanisms of muscle atrophy and hypertrophy: Implications in health and disease. Nat. Commun..

[4] Chal J., Pourquie O. (2017). Making muscle: Skeletal myogenesis in vivo and in vitro. Development.

[5] Feng L.T., Chen Z.N., Bian H. (2024). Skeletal muscle: Molecular structure, myogenesis, biological functions, and diseases. MedComm.

[6] Buckingham M., Rigby P.W.J. (2014). Gene Regulatory Networks and Transcriptional Mechanisms that Control Myogenesis. Dev. Cell.

[7] Moujaber O., Stochaj U. (2020). The Cytoskeleton as Regulator of Cell Signaling Pathways. Trends Biochem. Sci..

[8] Jaalouk D.E., Lammerding J. (2009). Mechanotransduction gone awry. Nat. Rev. Mol. Cell Biol..

[9] Harris A.R., Jreij P., Fletcher D.A. (2018). Mechanotransduction by the Actin Cytoskeleton: Converting Mechanical Stimuli into Biochemical Signals. Annu. Rev. Biophys..

[10] Nowak S.J., Nahirney P.C., Hadjantonakis A.K., Baylies M.K. (2009). Nap1-mediated actin remodeling is essential for mammalian myoblast fusion. J. Cell Sci..

[11] Zhang T., Zaal K.J., Sheridan J., Mehta A., Gundersen G.G., Ralston E. (2009). Microtubule plus-end binding protein EB1 is necessary for muscle cell differentiation, elongation and fusion. J. Cell Sci..

[12] Nguyen M.T., Won Y.H., Kwon T.W., Lee W. (2022). Twinfilin-1 is an essential regulator of myogenic differentiation through the modulation of YAP in C2C12 myoblasts. Biochem. Biophys. Res. Commun..

[13] Nguyen M.T., Min K.H., Kim D., Park S.Y., Lee W. (2020). CFL2 is an essential mediator for myogenic differentiation in C2C12 myoblasts. Biochem. Biophys. Res. Commun..

[14] Guerin C.M., Kramer S.G. (2009). Cytoskeletal remodeling during myotube assembly and guidance: Coordinating the actin and microtubule networks. Commun. Integr. Biol..

[15] Nguyen M.T., Dash R., Jeong K., Lee W. (2023). Role of Actin-Binding Proteins in Skeletal Myogenesis. Cells.

[16] Vakhrusheva A.V., Murashko A.V., Trifonova E.S., Efremov Y.M., Timashev P.S., Sokolova O.S. (2022). Role of actin-binding proteins in the regulation of cellular mechanics. Eur. J. Cell Biol..

[17] Takahashi K., Nadal-Ginard B. (1991). Molecular cloning and sequence analysis of smooth muscle calponin. J. Biol. Chem..

[18] Shibukawa Y., Yamazaki N., Kumasawa K., Daimon E., Tajiri M., Okada Y., Ikawa M., Wada Y. (2010). Calponin 3 regulates actin cytoskeleton rearrangement in trophoblastic cell fusion. Mol. Biol. Cell.

[19] Daimon E., Shibukawa Y., Wada Y. (2013). Calponin 3 regulates stress fiber formation in dermal fibroblasts during wound healing. Arch. Dermatol. Res..

[20] Rami G., Caillard O., Medina I., Pellegrino C., Fattoum A., Ben-Ari Y., Ferhat L. (2006). Change in the shape and density of dendritic spines caused by overexpression of acidic calponin in cultured hippocampal neurons. Hippocampus.

[21] Shibukawa Y., Yamazaki N., Daimon E., Wada Y. (2013). Rock-dependent calponin 3 phosphorylation regulates myoblast fusion. Exp. Cell Res..

[22] Ebner N., Anker S.D., von Haehling S. (2020). Recent developments in the field of cachexia, sarcopenia, and muscle wasting: Highlights from the 12th Cachexia Conference. J. Cachexia Sarcopenia Muscle.

[23] Stevenson E.J., Koncarevic A., Giresi P.G., Jackman R.W., Kandarian S.C. (2005). Transcriptional profile of a myotube starvation model of atrophy. J. Appl. Physiol..

[24] Screen M., Raheem O., Holmlund-Hampf J., Jonson P.H., Huovinen S., Hackman P., Udd B. (2014). Gene expression profiling in tibial muscular dystrophy reveals unfolded protein response and altered autophagy. PLoS ONE.

[25] Provenzano P.P., Keely P.J. (2011). Mechanical signaling through the cytoskeleton regulates cell proliferation by coordinated focal adhesion and Rho GTPase signaling. J. Cell Sci..

[26] Watt K.I., Goodman C.A., Hornberger T.A., Gregorevic P. (2018). The Hippo Signaling Pathway in the Regulation of Skeletal Muscle Mass and Function. Exerc. Sport Sci. Rev..

[27] Fischer M., Rikeit P., Knaus P., Coirault C. (2016). YAP-Mediated Mechanotransduction in Skeletal Muscle. Front. Physiol..

[28] Dupont S., Morsut L., Aragona M., Enzo E., Giulitti S., Cordenonsi M., Zanconato F., Le Digabel J., Forcato M., Bicciato S. (2011). Role of YAP/TAZ in mechanotransduction. Nature.

[29] Qin S., Li C., Lu H., Feng Y., Guo T., Han Y., Zhang Y., Tang Z. (2024). Biology of Hippo signaling pathway: Skeletal muscle development and beyond. J. Integr. Agric..

[30] Dupont S. (2016). Role of YAP/TAZ in cell-matrix adhesion-mediated signalling and mechanotransduction. Exp. Cell Res..

[31] Panciera T., Azzolin L., Cordenonsi M., Piccolo S. (2017). Mechanobiology of YAP and TAZ in physiology and disease. Nat. Rev. Mol. Cell Biol..

[32] Deato M.D.E., Marr M.T., Sottero T., Inouye C., Hu P., Tjian R. (2008). MyoD targets TAF3/TRF3 to activate myogenin transcription. Mol. Cell.

[33] She Y., Li C., Jiang T., Lei S., Zhou S., Shi H., Chen R. (2021). Knockdown of CNN3 Impairs Myoblast Proliferation, Differentiation, and Protein Synthesis via the mTOR Pathway. Front. Physiol..

[34] Flemming A., Huang Q.Q., Jin J.P., Jumaa H., Herzog S. (2015). A Conditional Knockout Mouse Model Reveals That Calponin-3 Is Dispensable for Early B Cell Development. PLoS ONE.

[35] Nair V.A., Al-Khayyal N.A., Sivaperumal S., Abdel-Rahman W.M. (2019). Calponin 3 promotes invasion and drug resistance of colon cancer cells. World J. Gastrointest. Oncol..

[36] Maddala R., Mongan M., Xia Y., Rao P.V. (2020). Calponin-3 deficiency augments contractile activity, plasticity, fibrogenic response and Yap/Taz transcriptional activation in lens epithelial cells and explants. Sci. Rep..

[37] Nardone G., Oliver-De La Cruz J., Vrbsky J., Martini C., Pribyl J., Skladal P., Pesl M., Caluori G., Pagliari S., Martino F. (2017). YAP regulates cell mechanics by controlling focal adhesion assembly. Nat. Commun..

[38] Abramovici H., Gee S.H. (2007). Morphological changes and spatial regulation of diacylglycerol kinase-zeta, syntrophins, and Rac1 during myoblast fusion. Cell Motil. Cytoskeleton.

[39] Pan D. (2010). The hippo signaling pathway in development and cancer. Dev. Cell.

[40] Densham R.M., O’Neill E., Munro J., König I., Anderson K., Kolch W., Olson M.F. (2009). MST Kinases Monitor Actin Cytoskeletal Integrity and Signal via c-Jun N-Terminal Kinase Stress-Activated Kinase To Regulate p21Waf1/Cip1 Stability. Mol. Cell. Biol..

[41] Lin Q., Cao J., Yu J., Zhu Y., Shen Y., Wang S., Wang Y., Liu Z., Chang Y. (2023). YAP-mediated trophoblast dysfunction: The common pathway underlying pregnancy complications. Cell Commun. Signal..

[42] Aragona M., Panciera T., Manfrin A., Giulitti S., Michielin F., Elvassore N., Dupont S., Piccolo S. (2013). A mechanical checkpoint controls multicellular growth through YAP/TAZ regulation by actin-processing factors. Cell.

[43] Yang C., Zhu S., Feng W., Chen X. (2021). Calponin 3 suppresses proliferation, migration and invasion of non-small cell lung cancer cells. Oncol. Lett..

[44] Junghans D., Herzog S. (2018). Cnn3 regulates neural tube morphogenesis and neuronal stem cell properties. FEBS J..

