# Supplementary material for: Calponin 3 Regulates Myoblast Proliferation and Differentiation Through Actin Cytoskeleton Remodeling and YAP1-Mediated Signaling in Myoblasts
Source: Cells. 2025 Jan 18;14(2):142. doi: 10.3390/cells14020142 (PMC11764405; doi:10.3390/cells14020142)
Supplement: Supplementary file 1 [file cells-14-00142-s001.zip › Supplementary Figure S1.pdf]

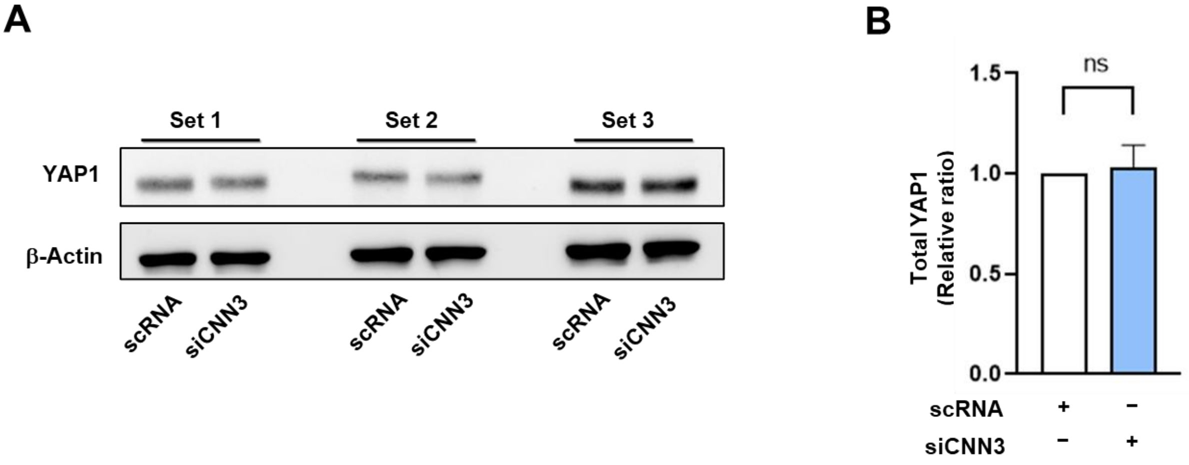

Figure S1. CNN3 knockdown did not alter YAP1 expression levels. C2C12 myoblasts were transfected with 200 nM of either control scRNA or siCNN3. **(A)** YAP1 and β-actin expression levels were assessed by immunoblotting 24 hours post-transfection. **(B)** YAP1 expression levels were normalized to β-actin, and relative ratios were calculated relative to the scRNA control. Data are presented as the mean ± SEM from three independent experiments, with "ns" indicating no significant difference.
