# Supplementary material for: Calponin 3 Regulates Myoblast Proliferation and Differentiation Through Actin Cytoskeleton Remodeling and YAP1-Mediated Signaling in Myoblasts
Source: Cells. 2025 Jan 18;14(2):142. doi: 10.3390/cells14020142 (PMC11764405; doi:10.3390/cells14020142)
Supplement: Supplementary file 1 [file cells-14-00142-s001.zip › Supplementary Table S1-S3.pdf]

Table S1. Oligonucleotide sequences for transfection

Gene	Oligonucleotide sequence (5´-3´)
scRNA (control RNA)	UCACAACCUCCUAGAAAGAGUAGA
siCNN3-1	GCAGAUGGGCACCAACAAAUU
siCNN3-2	AAUGAAGUUGCCGAUAUUC

Table S2. Primer lists and conditions for *q*RT-PCR

Gene	Primer sequence (5´-3´)		Product size	Annealing Temperature	Concentration		Cycle
					cDNA	Primer	
GAPDH	F.P	AACATCAAATGGGGTGAGGCC	252	58	2 ng/µl	0.5 µM	40
	R.P	GTTGTCATGGATGACCTTGGC					
PCNA	F.P	GAACCTGCAGAGCATGGACTC	201	58			
	R.P	GGTGTCTGCATTATCTTCAGCCC					
Cyclin D1	F.P	ACCAATCTCCTCAACGACCG	228	58			
	R.P	ACGGAAGGGAAGAGAAGGG					
Cyclin B1	F.P	GAGCTATCCTCATTGACTGG	125	58			
	R.P	CATCTTCTTGGGCACACAAC					

Table S3. Antibodies list

Antibody	Type	Targeted species	Manufacturer	Cat. No.	Dilution ratio*
CNN3	Monoclonal	Mouse	Santa Cruz Biotechnology, Dallas, TX, USA	sc-271188	1:5,000
MyHC	Monoclonal	Mouse	DSHB, Iowa, IA, USA	MF20	1:1,000
MyoD	Monoclonal	Mouse	Santa Cruz Biotechnology, Dallas, TX, USA	sc-377460	1:1,000
MyoG	Monoclonal	Mouse	Santa Cruz Biotechnology, Dallas, TX, USA	sc-12732	1:1,000
YAP1	Monoclonal	Rabbit	Cell Signaling Technology, Danvers, MA, USA	14074S	1:10,000
pYAP1	Polyclonal	Mouse	Cell Signaling Technology, Danvers, MA, USA	4911S	1:10,000
Lamin B2	Monoclonal	Rabbit	Abcam, Cambridge, United Kingdom	ab151735	1:2,500
α-tubulin	Monoclonal	Mouse	DSHB, Iowa, IA, USA	12G10	1:2,000
β-actin	Monoclonal	Rabbit	Sigma-Aldrich Chemical, St. Louis, USA	A2066	1:10,000
Antibodies HRP-linked anti-rabbit IgG			Cell Signaling Technology, Danvers, MA, USA	#7074	1:10,000
Goat anti-mouse(H+L)			Thermofisher Sci., Waltham, MA, USA	#32430	1:2,000

*All blots were visualized using a TOPview ECL Femto (Enzynomics).
